# *Odoribacter splanchnicus* inhibits toxin production in *Clostridioides difficile*: insights from clinical correlation and *in vitro* validation

**DOI:** 10.3389/fmicb.2026.1741232

**Published:** 2026-01-22

**Authors:** Na Wang, Jing Fan, Xianbo Geng, Shujuan Zhang, Zhaoyi Pan, Changzhong Jin, Yunbo Chen, Nanping Wu

**Affiliations:** 1 Jinan Microecological Biomedicine Shandong Laboratory, Jinan, China; 2 State Key Laboratory for Diagnosis and Treatment of Infectious Diseases, The First Affiliated Hospital, Zhejiang University School of Medicine, Hangzhou, China

**Keywords:** *Clostridioides difficile*, commensal bacteria, gut microbiome, *Odoribacter splanchnicus*, sporulation, toxin inhibition

## Abstract

**Background:**

*Clostridioides difficile* infection (CDI) is a leading cause of healthcare-associated diarrhea. Although gut microbiota dysbiosis is central to CDI, the specific commensal species that confer protection are not well defined.

**Methods:**

We performed 16S rRNA sequencing on fecal samples from a clinical cohort of 30 CDI patients, 30 non-CDI diarrhea patients, 27 asymptomatic *C. difficile* carriers, and 30 healthy controls. To functionally validate the clinical finding, an in vitro anaerobic co-culture system was established between the *Odoribacter splanchnicus* type strain and *C. difficile*. Toxin protein levels in the supernatant were quantified by ELISA at multiple time points (24, 48, and 72 h). Sporulation was assessed via ethanol resistance assays, and the expression of toxin genes (tcdA/tcdB) was measured by quantitative PCR (qPCR).

**Results:**

Clinical analysis revealed a significant negative correlation between the abundance of Odoribacter splanchnicus and CDI severity. In vitro, a high initial ratio of *O. splanchnicus* significantly suppressed *C. difficile* toxin production during the stationary phase, without inhibiting bacterial growth. This reduction *in vitro* levels was accompanied by a concurrent increase in sporulation and was preceded by a downregulation of tcdB gene expression.

**Conclusion:**

This work positions *O. splanchnicus* as a highly promising candidate for the development of next-generation, defined microbial therapeutics and provides a mechanistic foundation for future anti-virulence approaches to combat CDI.

## 1 Introduction

*Clostridioides difficile* is a Gram-positive, spore-forming, obligate anaerobe that often residing in human gut, capable of causing *C. difficile* infection (CDI) upon dysbiosis of gut microbiome (Smits et al., 2016; Kordus et al., 2022). The pathogenesis and clinical severity of CDI are primarily driven by the production of two large clostridial toxins: Toxin A (TcdA) and Toxin B (TcdB) (Voth and Ballard, 2005). These toxins are the primary virulence factors responsible for the hallmark symptoms of CDI including mild diarrhea, pseudomembranous colitis and toxic megacolon (Ofosu, 2016). In the past decades, CDI has become the leading cause of nosocomial gastrointestinal infection, with incidence rates increasing worldwide, resulting in high morbidity and mortality especially among hospitalized patients (Finn et al., 2021; Schnizlein and Young, 2022).

The management of CDI remains a major clinical challenge, primarily due to the multidrug-resistant nature of the pathogen. Current first-line therapies still rely on antibiotics such as vancomycin, metronidazole, and fidaxomicin (Gupta et al., 2018). However, after more than three decades of use, declining susceptibility to these agents has led to reduced cure rates and the emergence of multidrug-resistant strains (Caballero et al., 2017). Moreover, antibiotics like vancomycin and metronidazole further disrupt the gut ecological balance, resulting in high recurrence rates of 25–60% after initial treatment (Henson, 2021). Given these limitations, non-antibiotic approaches—particularly those targeting the microbiome—have regained significant research interest. Fecal microbiota transplantation (FMT) has emerged as an effective intervention for recurrent and refractory CDI by restoring microbial diversity and colonization resistance (Kelly et al., 2021; Khoruts and Staley, 2025). Nevertheless, FMT carries potential risks, including the transfer of antibiotic resistance genes, inadvertent transmission of pathogens, bacterial translocation, and long-term metabolic complications (Hudson et al., 2017; DeFilipp et al., 2019; DeLeon et al., 2025). One promising direction is developing defined microbial consortia or specific strains with known functions as an alternative to crude fecal suspensions, allowing more targeted and reproducible restoration of gut flora (Murphy et al., 2023; Yadegar et al., 2024). Given this, the identification of key commensal species that are directly associated with CDI progression, coupled with a mechanistic understanding of their function, is essential for guiding the development of targeted microbiota-based therapies.

Multiple studies have demonstrated that both community membership and abundance within the microbiota in patients with CDI are different from those of healthy individuals, including the lower richness and diversity, and decreased relative abundances of *Faecalibacterium*, *Roseburia*, *Bifidobacterium*, *Blautia*, Ruminococcaceae, Lachnospiraceae and other gut commensal members (Kachrimanidou and Tsintarakis, 2020; Vakili et al., 2020; Martinez et al., 2022; Piccioni et al., 2022). Nonetheless, targeted research aimed at identifying species directly linked to the clinical progression of CDI in patients remains scarce. Therefore, moving beyond general ecological observations to pinpoint clinically relevant species and delineate their precise functions has been of great significance to clinical applications.

In this study, we utilized 16S rRNA sequencing to characterize the gut microbiota alteration of hospitalized patients with or without CDI. Specifically, we focused on the asymptomatic colonization and CDI groups, aiming to identify the bacterial species whose abundance correlates with disease severity. Our analysis revealed that the abundance of *Odoribacter splanchnicus* exhibited a significant negative correlation with CDI severity. This bacterium is strictly anaerobic, Gram-negative, non-sporulating, known for its production of short-chain fatty acids (SCFAs), properties that make it a compelling candidate for development as a next-generation probiotic (Sato et al., 2021; Li et al., 2025). We demonstrate that *O. splanchnicus* directly suppresses the production of the key virulence factors, TcdA and TcdB, and exerts a multifaceted impact on the *C. difficile* life cycle, including its growth and sporulation by *in vitro* experiments. Our results may help to elucidate the protective role of commensal bacteria against *C. difficile* pathogenesis and pave the way for developing *O. splanchnicus*-based biotherapeutics for CDI treatment.

## 2 Materials and methods

### 2.1 Study design and specimen collection

Our study enrolled 117 patients hospitalized at the First Affiliated Hospital, Zhejiang University School of Medicine (China), after informed consent. Considering patients were hospitalized for different causes and subsequently underwent different treatments, no exclusion criteria except minimum age of 18 was used in this study. Stool samples collected from these 117 patients were divided into four groups associated with diarrhea and *C. difficile* colonization statuses: (1) Control group (CN, *n* = 30): patients without diarrhea symptoms and a negative *C. difficile* test by glutamate dehydrogenase (GDH), enzyme immunoassay (EIA) toxin A/B and quantitative PCR for toxin B gene. (2) Non-CDI diarrhea (NCD, *n* = 30): patients with diarrhea (a passage of more than 3 times of unformed stools) and a negative *C. difficile* test same as the control group. (3) CDI group (CDI, *n* = 30): patients diagnosed with new-onset diarrhea and a positive *C. difficile* test. (4) Asymptomatic colonization group (AC, *n* = 27): patients without diarrhea and a positive *C. difficile* test. Diarrhea was defined as ≥3 loose stools in 24 h (Vázquez-Cuesta et al., 2023). Patients who had CDI episodes or CDI treatment within the past 3 months were excluded.

Stool samples were collected in sterile containers and immediately transported to the laboratory for CDI diagnosis, and then stored at −80 °C until DNA extraction.

### 2.2 Fecal DNA extraction and quantitative PCR for toxigenic *Clostridioides difficile*

Stool DNA was extracted using the DNeasy PowerSoil Pro Kit (Qiagen, Germany) according to the manufacturer’s instructions. DNA concentration and purity were measured using NanoDrop 2000 (Thermo Fisher Scientific, MA, United States).

Primer sequences used for toxigenic *C. difficile* quantification were F: 5’-AGC AGTTGAATATAGTGGTTTAGTTAGAGTTG-3’and R: 5’-CATGCTTTTTTAGTTT CTGGATTGAA-3′ (Maestri et al., 2022). Quantitative PCR (qPCR) was performed on a C1000-Touch Real-time PCR instrument (Bio-Rad, United States). The reaction system and conditions of qPCR were established by following the protocol of ChamQ SYBR qPCR Master Mix (Vazyme, China). All samples were performed in duplicate, and samples were considered positive when both replicates were positive.

### 2.3 Bacterial 16S rRNA sequencing and raw data processing

The hypervariable V3V4 region of bacterial 16S rRNA gene was amplified using 343F (5’-TACGGRAGGCAGCAG-3′) and 798R (5’-AGGGTATCTAATCCT-3′). Amplicons were tested by 2% agarose gel electrophoresis and purified with Agencourt AMPure XP beads (Beckman Coulter Co., United States). The purified DNA samples were sent for sequencing on Illumina NovaSeq6000 platform (Shanghai OE Biotech, China). Raw sequencing data were deposited in the China National Center for Bioinformation (CNCB) under accession number PRJCA050088.

The raw sequencing data were trimmed and filtered to obtain high-quality data (clean data). Then amplicon sequence variants (ASV) and the ASV abundance table were generated using DADA2 with the default parameters of QIIME2 (Callahan et al., 2016). All ASVs were annotated and blasted against SILVA 138 database to obtain taxonomic classification.

### 2.4 Bioinformatic analysis

The core microbiota of each group based on ASV level was identified here. The richness of gut bacterial communities in CN, NCD, CDI and AC groups were estimated by Chao1 index. Principal coordinates analysis (PCoA) was generated to assess bacterial community structure among different groups based on unweighted unifrac distances. Co-occurrence network analyses were performed using the Molecular Ecological Network Analyses (MENA, http://ieg4.rccc.ou.edu/MENA/), and the network was visualized using Gephi 0.10.1 (Deng et al., 2012). We use linear discriminant analysis (LDA) and LDA effective size analysis (LEfSe) to illustrate species difference at the level of genus between AC and CDI groups. Spearman’s correlation analysis was applied to analyze the correlation between differential genus and disease severity. CDI severity score index was calculated in accordance with previous studies (Peretz et al., 2016; Mulki et al., 2017). For each patient the score incorporated nine parameters, each variable added one point: serum albumin <3 g/dL, a WBC count that is markedly high (>50,000 cells/mL) or low (<2000 cells/mL), serum creatinine >130 μmol/L, serum lactate levels >2.2 mmol/L, temperature >38.5 °C, systolic blood pressure <100 mmHg, altered mental status, severe abdominal tenderness or distention, intestinal symptoms such as ileus, obstruction, perforation, toxic mega-colon.

### 2.5 Bacteria strains and co-culture systems construction

*C. difficile* strain (CD) used for co-culture was clinical isolate (sequencing type 54, *tcdA*+/*tcdB*+) that was preserved in our laboratory. *O. splanchnicus* strain ATCC 29572 (OS) was purchased from BeNa Culture Collection, Henan, China. CD and OS were grown at 37 °C in brain-heart-infusion broth (Oxoid) supplemented with 0.5% yeast extract (Oxoid) and 0.1% L-cysteine (Sigma-Aldrich) (BHIS), under the anaerobic environment (80% nitrogen, 10% hydrogen, 10% carbon dioxide) (Smith et al., 2022).

To construct co-culture systems, single colonies of CD and OS were separately inoculated into BHIS and grown anaerobically at 37 °C overnight. Each strain was sub-cultured into fresh media and grown to mid-exponential phase (OD_600nm_ = 0.3 to 0.7). Then, the co-cultures of the two strains were prepared at different inoculation ratios (CD: OS = 9:1, CD: OS = 1:1, CD: OS = 1:9), and the final inoculum concentration was 1 × 10^7^ CFU/mL.

### 2.6 Determination of co-culture proportions by species-specific qPCR

At each timepoint (6 h,12 h,18 h,24 h and 30 h), 1 mL samples of co-cultures were taken, centrifugated at 12000 g for 1 min and the supernatant discarded. Cell pellets were frozen at −80 °C until gDNA extractions were performed. The gDNA was extracted using a DNeasy PowerSoil Pro Kit (Qiagen) as per the manufacturer’s instructions. Primers specific to OS were designed based on the sequence of the hypervariable regions of 16S rRNA gene (F: 5′- GTGGGTAGCGAACAGGATTAG-3′; R: 5′- CCCAGGTGGCTCACTTAATAC-3′). Primers specific to CD and the qPCR reaction was performed as mentioned above. For absolute quantitation of copy numbers of CD and OS, a standard curve method was employed. Briefly, a fragment containing the target qPCR amplicon for each bacterium was amplified from genomic DNA using specific primers and cloned into a standard plasmid vector using a TA cloning kit (Promega, United States). The resulting recombinant plasmids were verified by sequencing. Plasmid DNA was extracted and quantified using a spectrophotometer. The copy number of each plasmid per microliter was calculated and a ten-fold serial dilution series of each plasmid was prepared to generate the standard curve. The amplification efficiency was 105.9%, *R*^2^ = 0.9995 for OS primers and 107.8%, *R*^2^ = 0.9979 for CD primers (Supplementary Figure S1). The copy numbers of CD and OS in each experimental sample were then interpolated from their respective standard curves.

### 2.7 Detection of toxin production in co-cultures by ELISA

Toxin production was monitored over time by collecting culture supernatants at 12-, 24-, 48-, and 72-h post-inoculation. Toxins (both TcdA and TcdB) concentrations in co-cultures were determined using ELISA kit (tgcBiomics, Germany). The blank media used for culturing was set as negative control, and supernatant of CD monocultures was set as positive control (Sulaiman et al., 2024).

### 2.8 Relative quantification of *tcdA* and *tcdB* expression

For gene expression analysis, 1 mL aliquots of the co-culture were harvested at 12, 24, 48, and 72 h. Bacterial cells were pelleted by centrifugation at 12,000 g for 1 min. Total RNA was then extracted from the pellet using the RNeasy mini kit (Qiagen, Germany) with on-column DNase I digestion to eliminate genomic DNA contamination. The isolated total RNA was reverse transcribed by PrimeScript™RT Master Mix (Takara, Japan) according to manufacturer’s instructions. A 7500 real-time PCR system (Bio-Rad, United States) was used to perform quantitative PCR reactions. The system and conditions of RT-qPCR were established by following the protocol of the SYBR^®^ Premix Ex Taq Kit (Takara, Japan). Primer sequences used for *tcdA* quantification were tcdA-F: 5’-GCGGAAATGGTAGAAATG-3′ and tcdA-R: 5’-ATCAGGTGCTATCAATACTT-3′ (Zhu et al., 2021). Primer sequences used for *tcdB* quantification were F: 5’-AGC AGTTGAATATAGTGGTTTAGTTAGAGTTG-3’and R: 5’-CATGCTTTTTTAGTTT CTGGATTGAA-3′ (Maestri et al., 2022). 16 s rRNA of *C. difficile* used as an internal control, primer sequences were CD16-F: 5’-TTGAGCGATTTACTTCGGTAAAGA-3′ and CD16-R: 5’-CCATCCTGTACTGGCTCACCT-3′. Relative gene transcript levels was calculated by the 2^−ΔΔCT^ method (Livak and Schmittgen, 2001). All reactions were carried out in triplicate.

### 2.9 Cytotoxicity assay

Cytotoxicity assays were done on Vero cells (Trzilova et al., 2022). For the assay, Vero cells were passaged in a 96-multiwell plate and incubated for 24 h prior to use. Cells were seeded in a six-well plate (3 × 10^5^ cells/well), in Dulbecco’s Modified Eagles Medium (DMEM), supplemented with 10% fetal bovine serum. Cells were grown for 24 h at 37 °C, 5% CO_2_ in air atmosphere. Supernatants collected at various time points were centrifuged and filtered through a 0.22 μm filter. 50 μL of the final content was added to each well plate and plates were incubated at 37 °C, in 5% CO_2_, for 18 h. Empty BHIS medium served as negative control. Cell rounding was assessed using a 10x objective on a light microscope.

### 2.10 Ethanol resistance sporulation assay

Ethanol resistance was used to determine the sporulation efficiency of monoculture and co-culture systems based on previously described procedures (Edwards and McBride, 2014; Donnelly et al., 2022). 0.5 mL aliquot of culture was mixed with 0.5 mL 95% ethanol, subjected to vortex mixing, incubated at room temperature for 15 min, serially diluted in 1 × PBS, and plated on BHIS with 0.1% taurocholate to enumerate spores.

### 2.11 Statistical analyses

Our monoculture and co-culture growth contain 3 biological replicates, data are expressed as mean ± SEM. Statistical analysis among multiple groups was performed using SPSS (24.0) for one-way ANOVA analysis, and statistical significance was set at *p* < 0.05. These results were visualized Origin 2024 software.

## 3 Results

### 3.1 Participant characteristics

The 117 patients included in our study were divided into the control group (CN, 30 subjects), non-CDI diarrhea group (NCD, 30 subjects), CDI group (CDI, 30 subjects) and asymptomatic carrier group (AC, 27 subjects). The patients’ characteristics are presented in Table 1. The median age of all group patients was around of 60 years, and the majority of AC and CDI groups were long stay inpatients (over 30 days). There were no significant differences in the rate of use of antibiotics among groups (*p* = 0.348).

**Table 1:** Characteristics of patients.

Characteristics	CN (*n* = 30)	NCD (*n* = 30)	CDI (*n* = 30)	AC (*n* = 27)	*P*
Age					
Mean ± SD	59.33 ± 16.86	58.73 ± 13.56	63.77 ± 17.04	65.33 ± 18.48	0.345
Sex					
Female	11 (36.7%)	15 (50%)	9 (30%)	12 (44.4%)	0.458
Male	19 (63.3%)	15 (50%)	21 (70%)	15 (55.6%)	
Hospital stays					
≤30	9 (30%)	18 (60%)	4 (13.3%)	6 (22.2%)	
>30 – ≤60	1 (3.3%)	5 (16.7%)	7 (23.3%)	3 (11.1%)	
>60 – ≤90	7 (23.3%)	1 (3.3%)	4 (13.3%)	3 (11.1%)	
>90	13 (43.3%)	6 (20%)	15 (50%)	15 (55.6%)	
Ward at stool collection					
With cancer	3 (10%)	7 (23.3%)	6 (20%)	4 (14.8%)	
With hematopathy	10 (33.3%)	4 (13.3%)	1 (3.3%)	5 (16.7%)	
With respiration failure	2 (6.7%)	0 (0%)	1 (3.3%)	2 (7.4%)	
With renal insufficiency	1 (3.3%)	0 (0%)	4 (13.3%)	0 (0%)	
Antibiotics usage					
Yes	26 (86.7%)	21 (70%)	21 (70%)	23 (85.2%)	0.348

### 3.2 Bacterial community structure analysis

**Figure 1:**
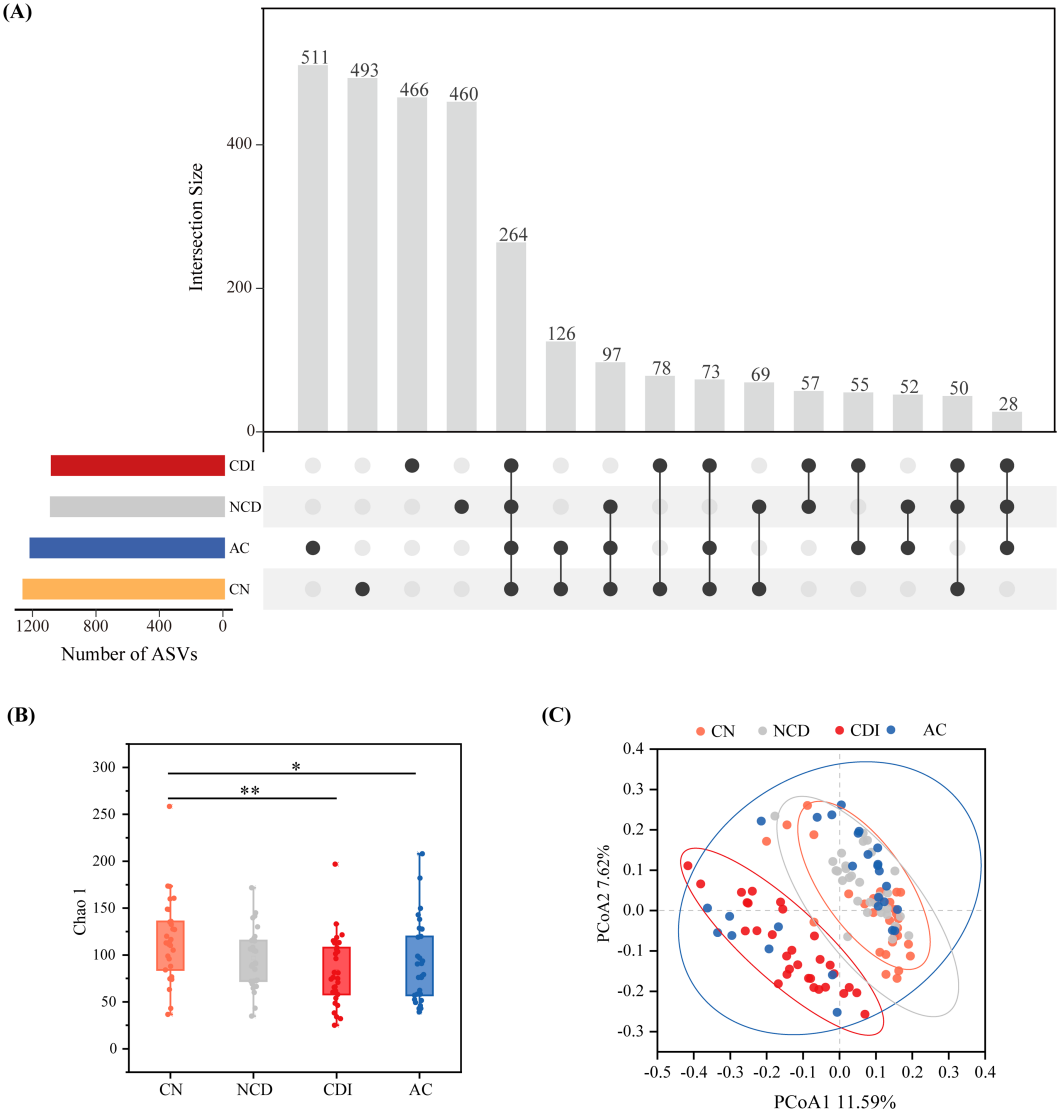
Biodiversity of gut microbiota from CN, NCD, CDI and AC groups. **(A)** Upset plot showing shared and unique ASVs of CN, NCD, AC and CDI groups. Calculations are based on ASV abundance table. **(B)** α-diversity comparison between the groups via Chao index analysis. * *p* < 0.05, ** *p* < 0.01. **(C)** *β*-diversity comparison via unweighted UniFrac distances PCoA analysis.

**Figure 2:**
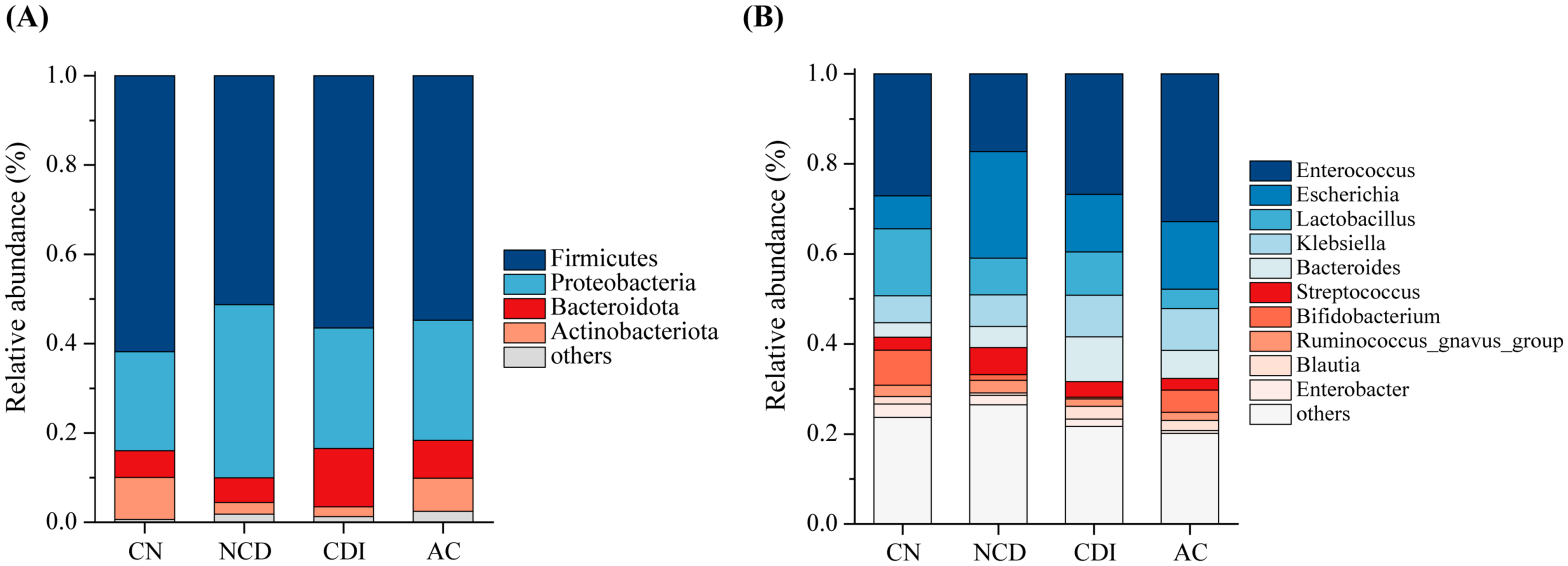
Bar plot of the taxonomic composition of the microbial community at the phylum level **(A)** and genus level **(B)** for CN, NCD, CDI and AC groups.

In total, 2,879 ASVs were obtained from the 117 fecal samples, and the number of ASVs of each sample ranged from 25 to 258. According to previous studies, the ASVs that presented in all groups were defined as core taxa, the ASVs that only presented in one group were defined as unique taxa, and the ASVs shared by two or three groups were defined as other taxa (Shade and Handelsman, 2012). As shown in Figure 1, the numbers of total ASVs were similar between CN (1,250 ASVs) and AC (1,206 ASVs) groups, while the total ASV numbers of NCD (1,077 ASVs) and CDI (1,071 ASVs) groups were very close. The number of ASVs that were shared by all groups was 264, and the four groups had large proportion of unique ASVs, and the number of unique ASVs was highest in AC (511 ASVs), followed by CN (493 ASVs), CDI (466 ASVs) and NCD (460 ASVs). The shared ASVs in CN&AC were highest (126 ASVs), followed by CN&AC&NCD (97 ASVs). The *α*-diversity analysis suggested that the microbial diversity was significantly decreased in CDI and AC groups, compared to CN group (Figure 2A). Furthermore, *β*-diversity analysis indicated that the composition of bacterial communities in CDI group significantly differed from other groups (PERMANOVA, *p* = 0.001), while CN, NCD and AC groups had similar microbial community structure (Figure 2B). Altogether, these findings indicated patients with true infection had unique gut microbial community, and the gut microbiome may be a potential biomarker source.

To further understand the bacterial composition differences among CN, NCD, CDI and AC groups, we analyzed the relative abundance of fecal microbes in each group at phylum and genus level, respectively. Generally, the phylum Firmicutes displayed the highest relative abundance in all groups (CN, 61.8%; NCD, 51.3%; CDI, 56.5%; AC, 54.7%) (Figure 2A). The phylum Proteobacteria was increased in CDI (27.0%), AC (27.0%), and especially in NCD (38.8%) compared to CN (22.2%). In addition, CDI and AC group had increased Bacteroidota and decreased Actinobacteriota at phylum level compared to CN and NCD groups. Moreover, the differences among groups at the genus level (top 10 in abundance) were further investigated. As shown in Figure 2B, the relative abundance of *Lactobacillus* and *Bifidobacterium* decreased in NCD, CDI and AC groups, while the relative abundance of *Escherichia*, *Klebsiella* and *Streptococcus* increased in comparison with CN group.

### 3.3 Microbial correlation network analysis

**Figure 3:**
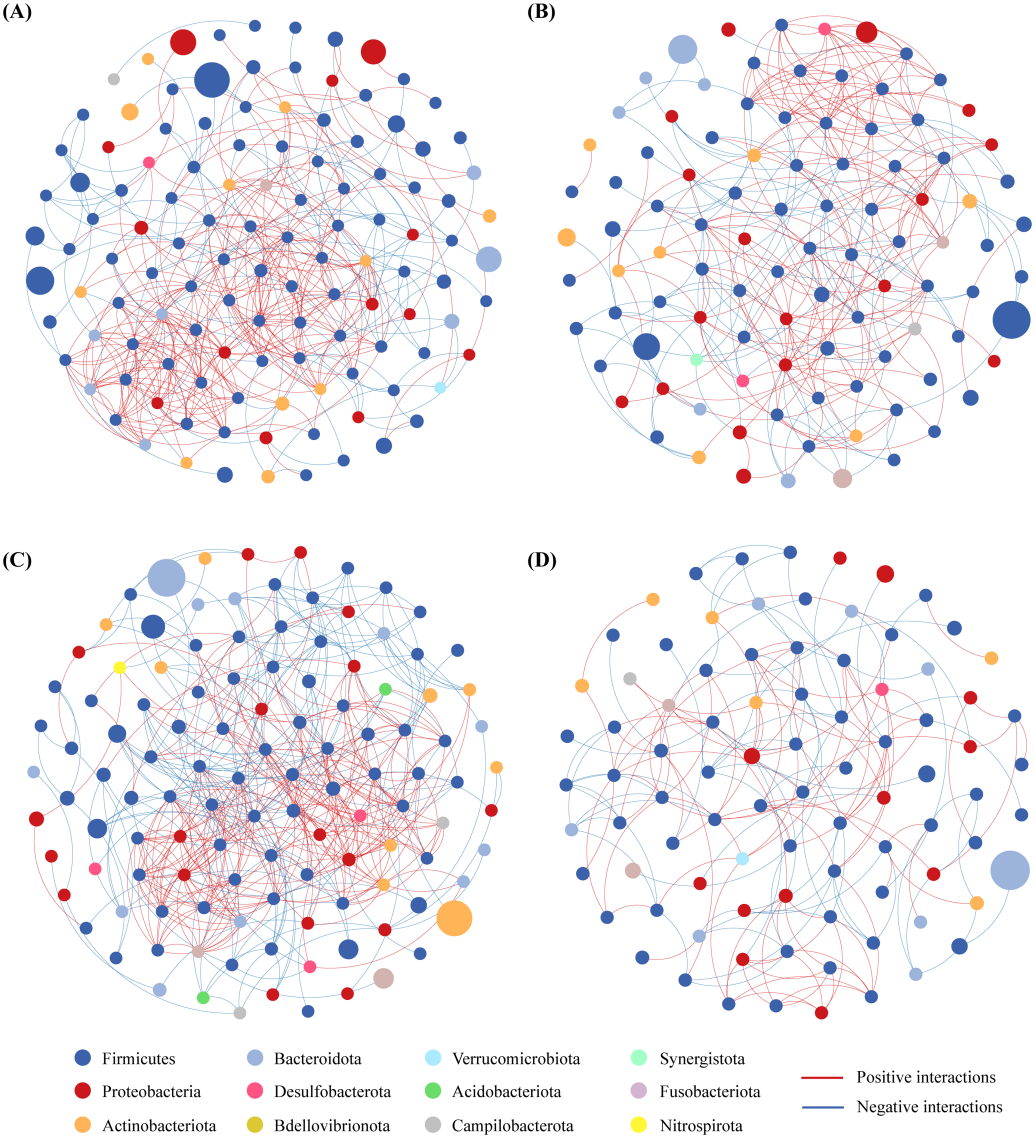
Genus co-occurrence networks of four groups based on Spearman correlation analysis. **(A)** CN group. **(B)** NCD group. **(C)** AC group. **(D)** CDI group. Each node represents a bacterial genus. The node size represents the relative abundance of each genus and the node color signifies the phylum with which the genus was affiliated. The red and blue lines refer to the Spearman coefficient, indicating positive and negative interactions between the nodes, respectively. Only high the high-confidence interactions (*p* < 0.05) with high absolute correlation coefficients (>0.63) were presented.

Furthermore, microbial co-occurrence network was constructed on genus level (>0.1% relative abundance) to show the interactions among individual species. The network analysis results highlighted the differences of interaction properties between the four groups (Figure 3). In comparison with CN (119 nodes, 398 edges), NCD (99 nodes, 292 edges) and CDI (88 nodes, 170 edges) had lower number of nodes and edges. Moreover, NCD and CDI also showed the decreasing average degree and lower proportion of negative interactions, indicating weaker bacterial interactions in these two groups. AC (117 nodes, 426 edges) had more edges and higher average degree, however, the proportion of negative interactions decreased in AC in comparison with CN. CDI had distinct network structure compared to the other groups, shown with lower number of nodes and edges, decreased average degree and higher modularity (Table 2). These results suggested that the bacterial interactions were much weaker in CDI group.

**Table 2:** The topological features of co-occurrence networks associated with different groups.

Network indexes	CN	NCD	AC	CDI
Total nodes	119	99	117	88
Total links	398	292	426	170
Average degree (avgK)	6.689	5.899	7.282	3.864
Average clustering coefficient (avgCC)	0.133	0.173	0.113	0.053
Average path distance (GD)	3.213	3.063	3.226	3.754
Modularity	0.51	0.487	0.404	0.538
Negative links	286	191	246	94
Positive links	112	101	180	76
Negative links/Total links	0.719	0.654	0.577	0.553

### 3.4 Biomarker identification and assessment

**Figure 4:**
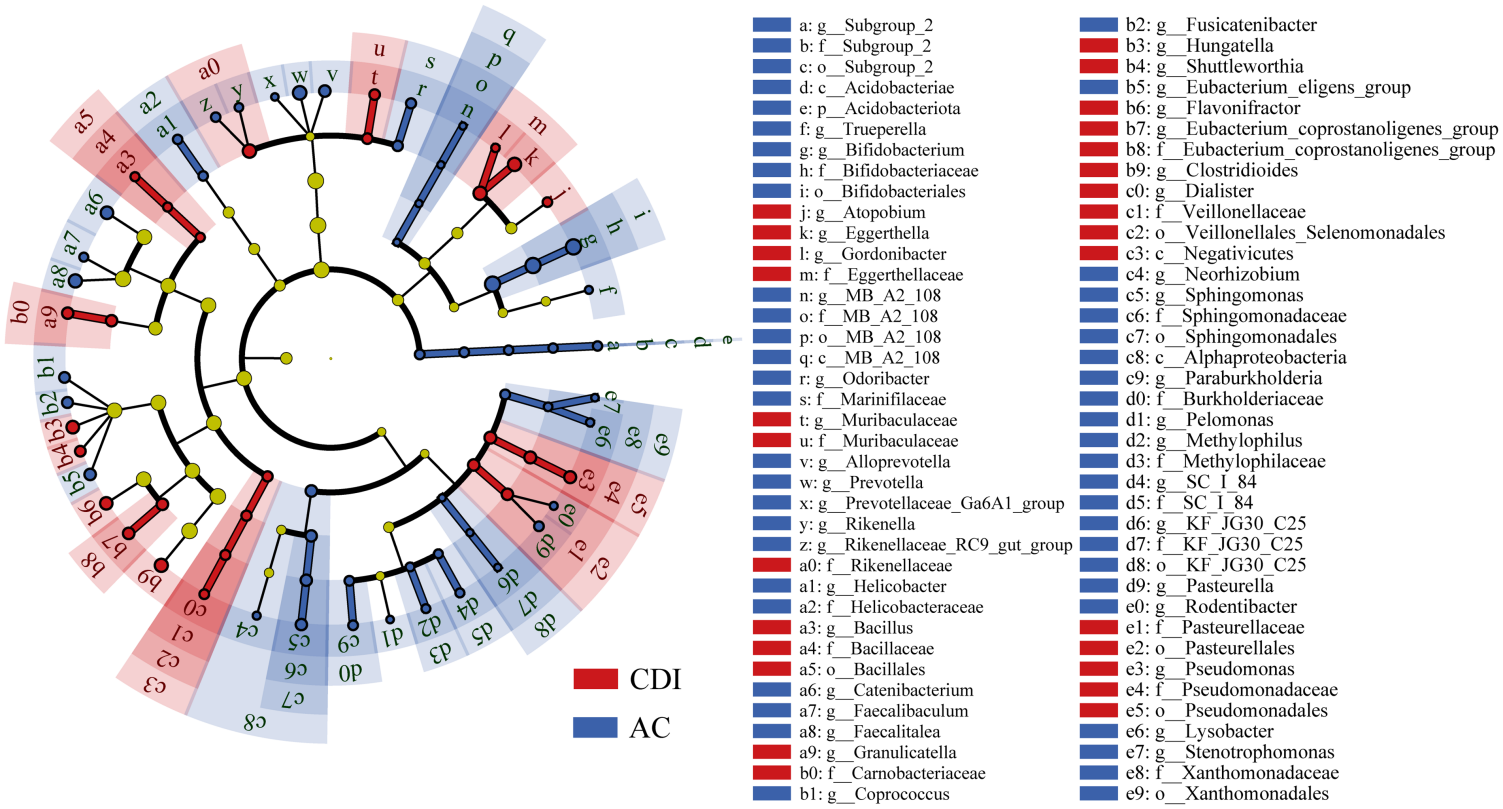
The cladogram of LEfSe analysis from phylum to genus between CDI and AC (LDA score > 2).

Bacterial community structure and network analysis had suggested the significant gut microbiota alteration in CDI patients. To explore the biomarkers that could distinguish the true infection from asymptomatic colonization, we explored the most discriminative bacteria between AC and CDI groups using LefSe analysis. The cladogram indicated 76 discriminative features between AC and CDI groups (LDA score ≥ 2, Figure 4). Some known and potential probiotics including *Bifidobacterium*, *Faecalibaculum* and *Odoribacter* were significantly decreased in CDI group, while *Dialister*, *Pasteurella*, *Pseudomonas* and Veillonellaceae were enriched in CDI group.

**Figure 5:**
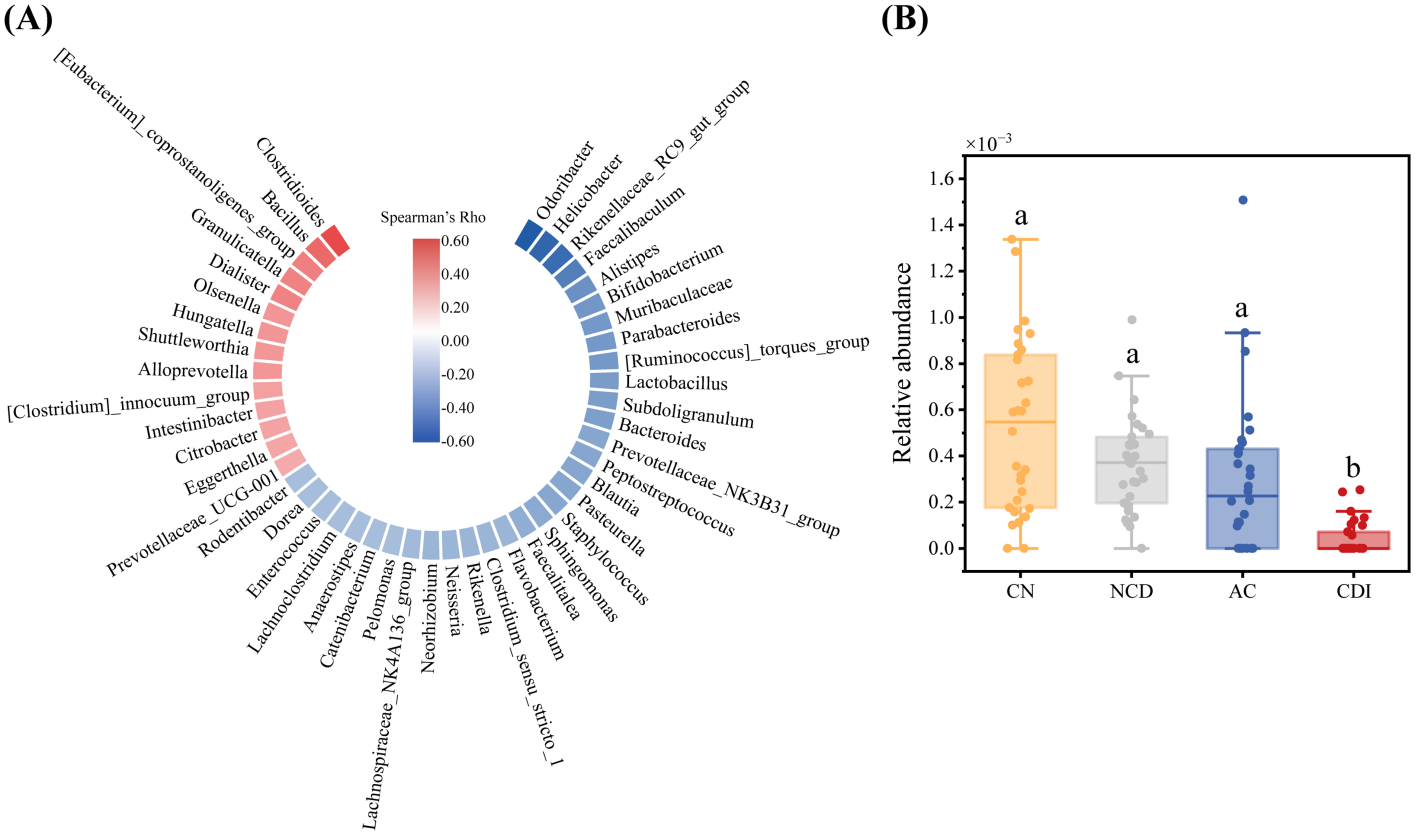
**(A)** Correlation analysis of discriminative genus and CDI severity. Red represents a positive correlation, blue represents a negative correlation, and only significant correlations (*p* < 0.05) were shown here. **(B)** Relative abundance of *Odoribacter* in CN, NCD, AC and CDI groups (The same letter represents no significant difference between the groups, different letters represent significant differences (*p* < 0.05) between the groups).

Next, all CDI patients were evaluated for the severity of the disease according to the criteria described above (Supplementary Figure S2), and genera associated with CDI disease severity were analyzed. In total, 46 genera were significantly associated with severity scores, of which 14 were positively correlated with severity scores and 32 were negatively correlated with severity scores (Figure 5A). Among them, *Odoribacter* was the most negatively correlated with CDI severity scores, suggesting it’s abundance may correlate with CDI disease progression. Subsequently, the abundance of *Odoribacter* among four groups was analyzed. Concordantly, we found that the abundance of *Odoribacter* was significantly lower in CDI group compared with CN, NCD and AC group (Figure 5B).

### 3.5 *Odoribacter* and *Clostridioides difficile* interactions in co-culture systems

**Figure 6:**
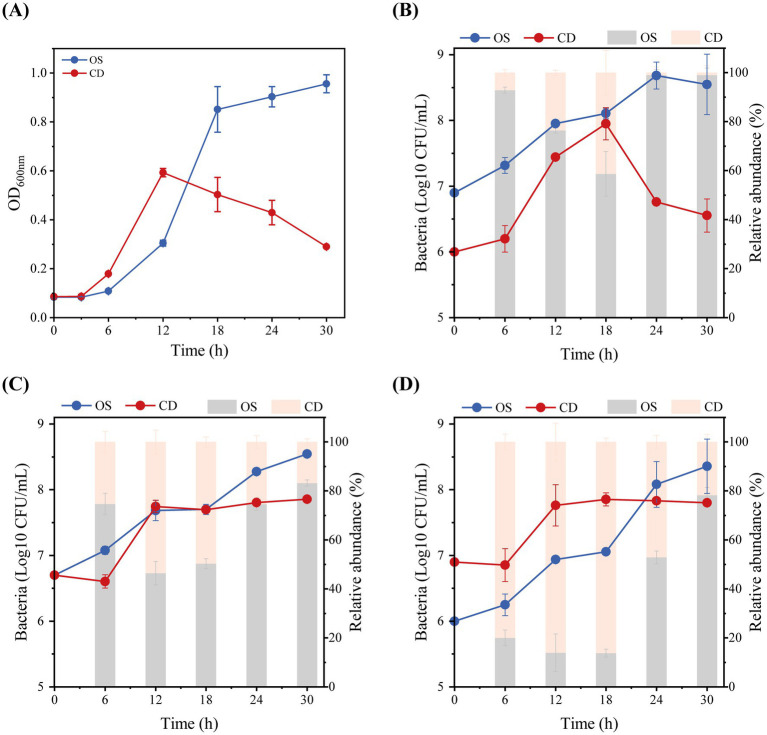
**(A)** Growth curves of CD and OS in monoculture. **(B)** Co-culture of CD and OS in the 1:9 ratio system. Bacteria load (left Y axis, line chart) and proportions of the two species (right Y axis, bar graph). **(C)** Co-culture of CD and OS in the 1:1 ratio system. **(D)** Co-culture of CD and OS in the 9:1 ratio system. Absolute quantitation for CD and OS were calculated from an absolute standard curve method using the plasmid template for each target gene. Values represent the mean of three biological replicates. Error bars represent the mean ± SEM of three biological replicates.

To determine whether *Odoribacter* influences key pathogenesis phenotypes of *C. difficile* (CD) including bacterial growth, sporulation, and toxin production, we selected the type strain *O. splanchnicus* ATCC 29572 (OS) and established CD-OS co-culture systems with different bacterial concentration ratio and quantified the proportions of the two strains. We first determined the growth kinetics of CD and OS in monoculture. The growth curve of OS was characterized by a prolonged lag phase compared with CD (Figure 6A). And we noticed the optical density of CD cultures began a sustained decrease after 12 h, which might be indicative of the initiation of sporulation. The results of co-culture systems showed that OS bacterial load finally stabilized at 5 × 10^8^ CFU/mL across all co-culture systems. However, the final bacterial load of *C. difficile* exhibited a significant reduction specifically in the 1:9 (CD: OS) ratio system (Figure 6B). In contrast, the CD load remained stable at around 7 × 10^7^ CFU/mL in the 1:1 and 9:1 ratio systems (Figures 6C,D). The shifting compositional ratio of the two species over time was also evaluated. The data show that the proportion of CD increased progressively until 18 h, after which it underwent a consistent decrease. This reversal ultimately resulted in the dominance of OS in all systems at 30 h (Figures 6B–D).

### 3.6 *Odoribacter* inhibit toxin production of *Clostridioides difficile* at high concentration

**Figure 7:**
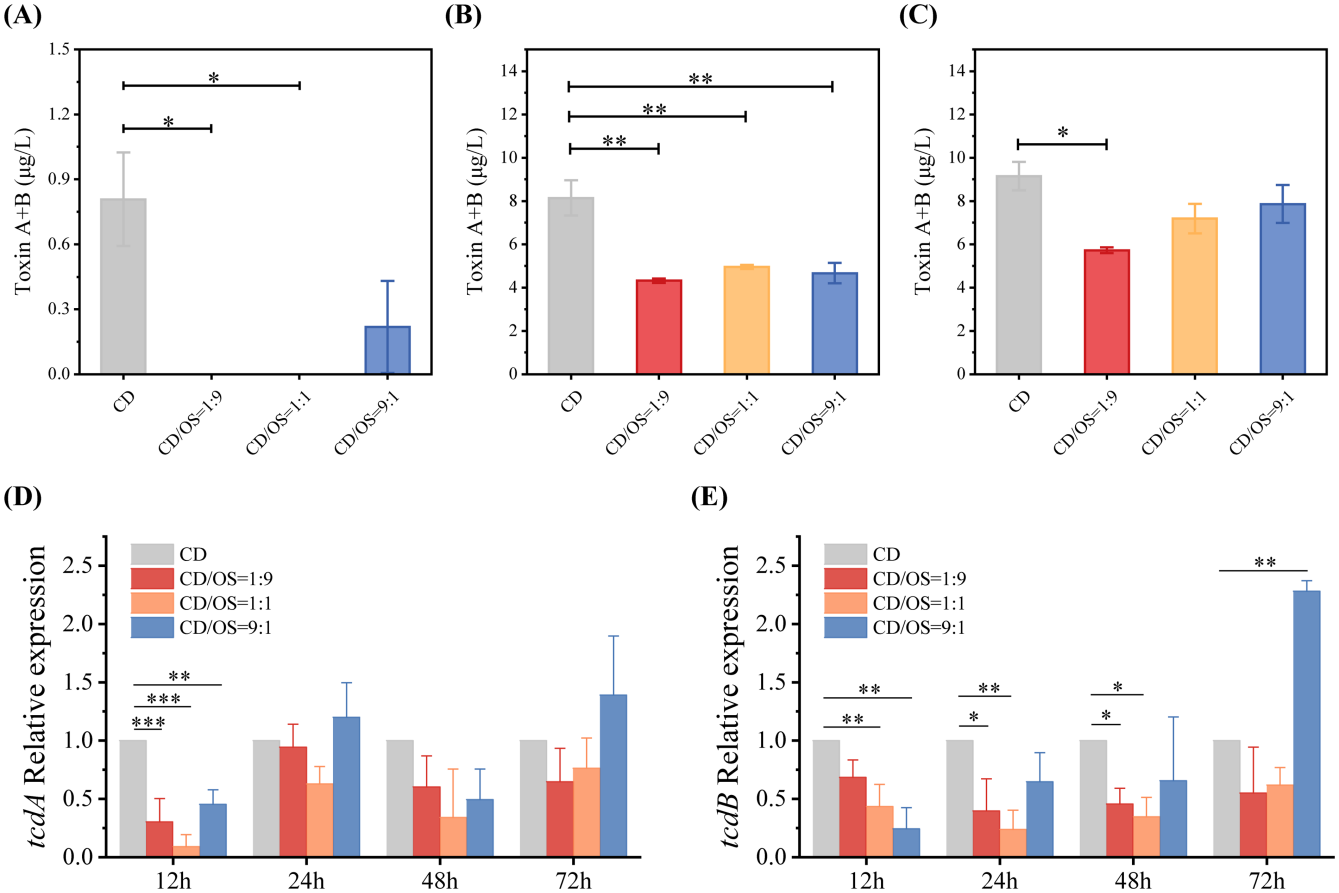
TcdA/TcdB production and *tcdA*/*tcdB* gene expression in CD monoculture and CD-OS co-cultures at different time points. **(A)** TcdA/TcdB production at 24 h. **(B)** TcdA/TcdB production at 48 h. **(C)** TcdA/TcdB production at 72 h. **(D)** Relative gene expression of *tcdA* at 12, 24, 48 and 72 h. **(E)** Relative gene expression of *tcdB* at 12, 24, 48 and 72 h. The relative quantitative gene expression was calculated with the use of 2^−ΔΔCT^ method. The relative expression levels of *tcdA/B* gene in CD monoculture were normalized to 1, with the 16S rDNA of CD used as an internal reference. All data were represented as mean ± SEM (*n* = 3). * *p* < 0.05, ** *p* < 0.01, *** *p* < 0.001.

We next investigated the impact of OS on toxin production by CD. A time-course analysis of toxin levels in culture supernatants was performed by ELISA. At the 12-h time point, toxin accumulation was not detectable in any culture group, with levels falling below the assay’s detection limit (data not shown). By 24 h, toxins were detected in the CD monoculture and the CD: OS = 9:1 co-culture, while it remained undetectable in the CD: OS = 1:1 and 1:9 groups (Figure 7A). A significant difference emerged at 48 h, where the toxin titer in the CD monoculture reached approximately 8 μg/L, which was significantly higher than in all co-culture conditions (Figure 7B). At 72 h, only the CD: OS = 1:9 co-culture maintained a statistically significant reduction in toxin levels compared to the CD monoculture. The toxin levels in the CD: OS = 9:1 and 1:1 groups, although numerically lower, were not significantly different from the monoculture control (Figure 7C).

To elucidate the molecular mechanism underlying this suppression, we quantified the expression of the toxin genes *tcdA* and *tcdB* via qPCR at corresponding time points. At the early 12-h time point, the expression of both *tcdA* and *tcdB* was significantly lower in all co-culture groups compared to the CD monoculture (Figures 7D,E). At subsequent time points, *tcdA* expression in co-cultures showed no significant difference from the monoculture. In contrast, a sustained transcriptional repression of *tcdB* was observed specifically in the CD: OS = 1:9 and 1:1 co-cultures at 24 h, where its expression remained significantly suppressed compared to the CD-alone control.

**Figure 8:**
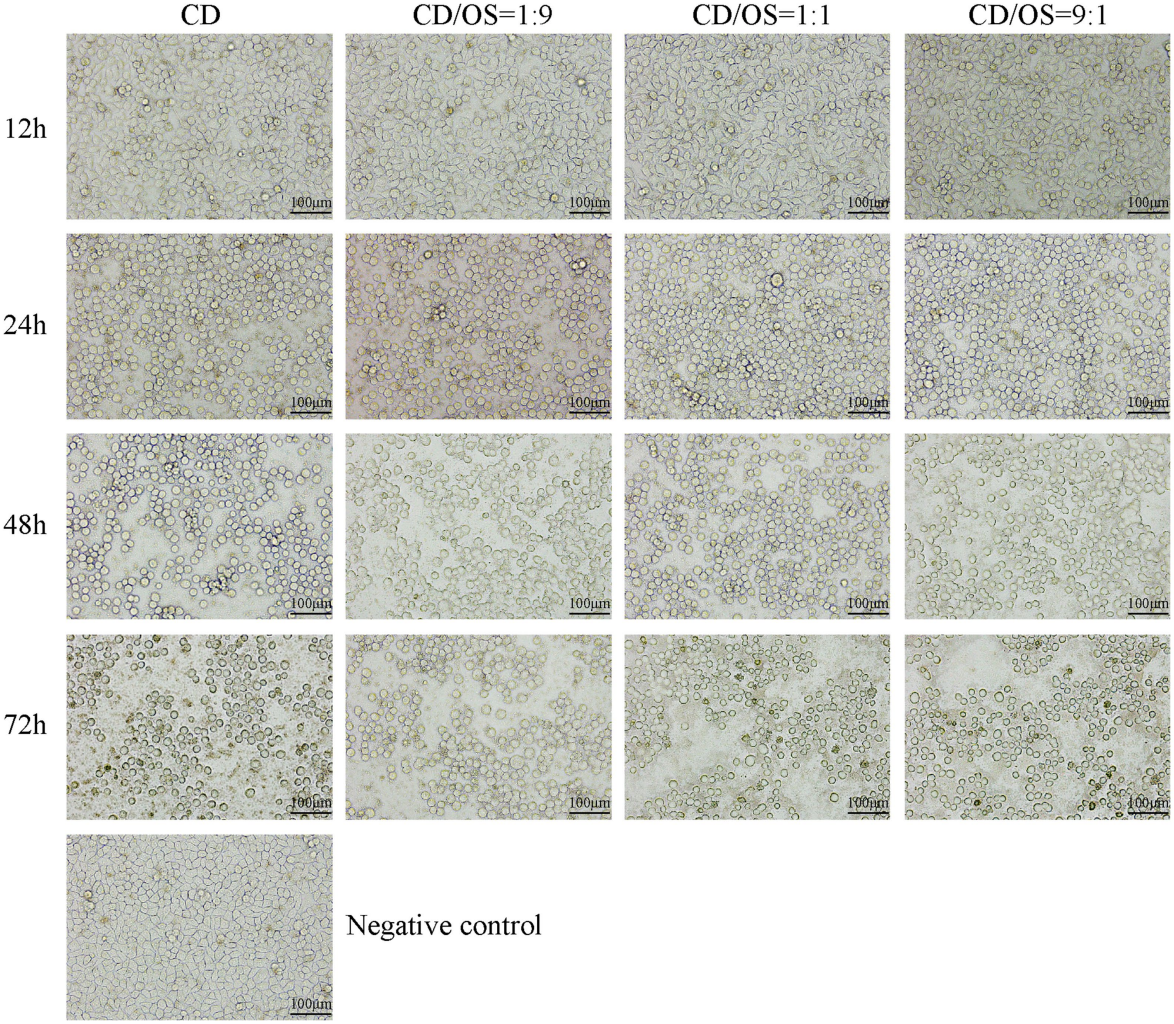
Cytopathic effects of co-culture supernatants collected from different time points on Vero cells. Each column represents a different group (from left to right, CD monoculture, CD/OS = 1:9, CD/OS = 1:1, CD/OS = 9:1). Each row represents a different time point (from up to down, 12 h, 24 h, 48 h, 72 h). Vero cells treated with empty BHIS medium were used as negative control, cells treated with supernatants from CD monoculture were used as positive control. Experiments were performed in triplicate and representative pictures were shown. Scale bar: 100 μm.

To validate that functional toxin was being released, a Vero cell cytotoxicity assay was performed using culture supernatants harvested at 12, 24, 48, and 72 h. Morphological analysis revealed a clear time-dependent and co-culture-dependent cytopathic effect (Figure 8). Supernatants from the 12-h time point, including that from the CD monoculture, induced no apparent morphological changes in Vero cells compared to the negative control. Exposure to 24-h supernatants resulted in initial cell rounding, which was markedly more severe in cells treated with the CD monoculture supernatant than in those treated with co-culture supernatants (CD: OS = 9:1, 1:1, and 1:9), where a greater proportion of cells remained adherent. Treatment with 48-h and, particularly, 72-h supernatants led to extensive cell rounding and detachment. Notably, cells exposed to the 72-h supernatant from the CD monoculture showed substantial death. Across these later time points, the cytopathic effect was consistently mildest in cells treated with the supernatant from the CD: OS = 1:9 co-culture.

### 3.7 *Odoribacter* promotes sporulation of *Clostridioides difficile* at earlier stage

**Figure 9:**
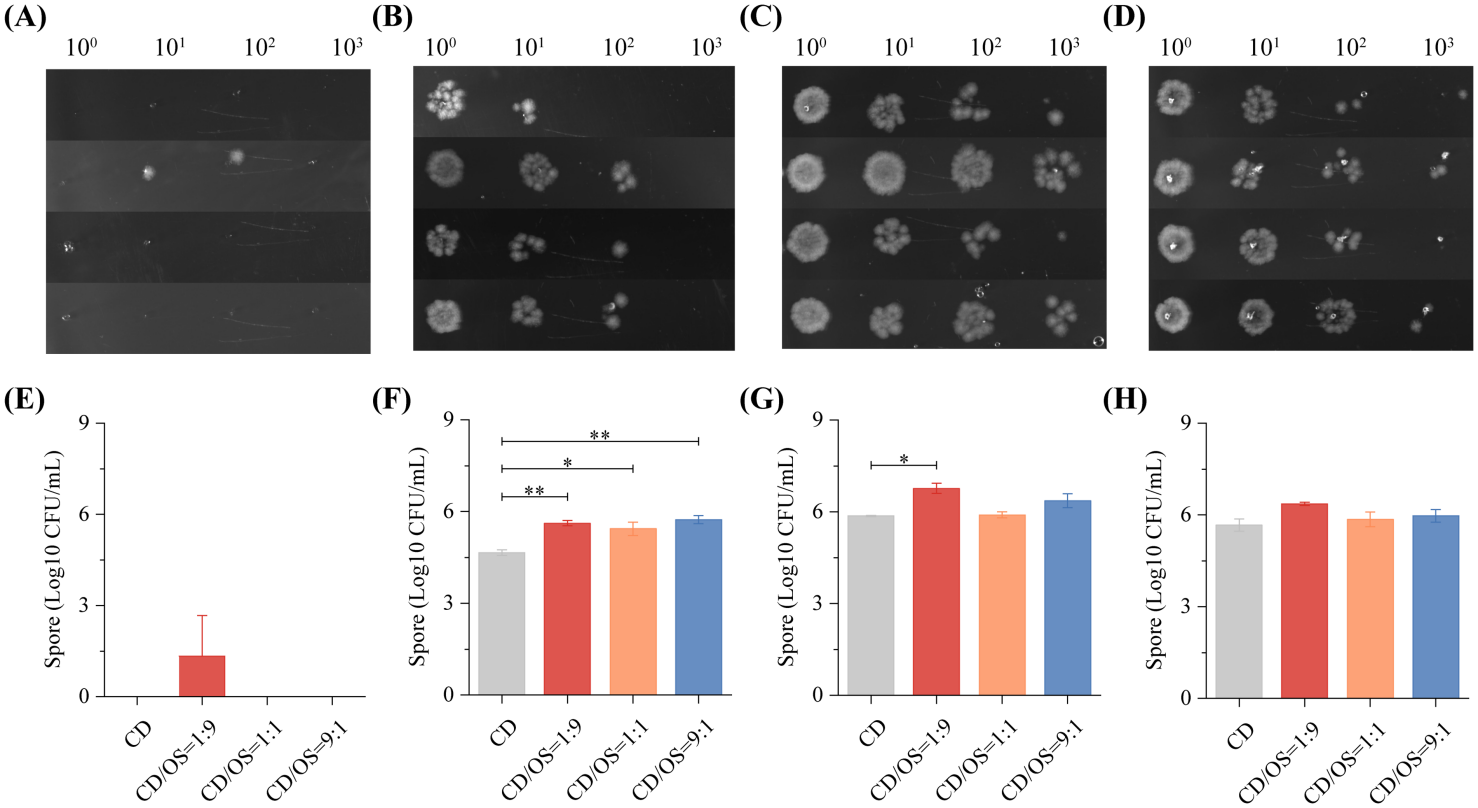
Spore quantification of CD monoculture and CD-OS co-cultures at different time points. **(A–D)** Colony-forming units (CFU) for spore quantification of CD monoculture and CD-OS co-cultures at 12, 24, 48 and 72 h, respectively. Each group was serially diluted in a 10-fold gradient, 1 μL of each dilution were spotted onto BHIS agar plates (supplemented with 0.1% taurocholate) and incubated anaerobically at 37 °C. Representative pictures were shown. **(E–H)** Statistics for spore production of CD monoculture and CD-OS co-cultures at different time points. All data were represented as mean ± SEM (*n* = 3). * *p* < 0.05, ** *p* < 0.01.

We further assessed the dynamics of CD spore formation under different co-culture conditions. Utilizing the ethanol tolerance of spores, we collected cultures at various time points, and quantified viable spores by plate counting. The results indicated that no significant spore formation was detected in any group at the early 12-h time point (Figures 9A,E). Although trace spores were observed in some replicates of the CD: OS = 1:9 group, the high variability between biological replicates precluded statistical significance. By 24 h, the spore counts in all co-culture groups (CD: OS = 9:1, 1:1, and 1:9) were significantly higher than in the CD monoculture (Figures 9B,F). At the subsequent 48-h and 72-h time points, total spore numbers increased across all groups, with the CD: OS = 1:9 co-culture consistently yielding the highest spore count (Figures 9C,D,G,H).

## 4 Discussion

The association between gut microbiota dysbiosis and *Clostridioides difficile* infection (CDI) is well-established. Consistent with prior research, we also found CDI patients exhibited a marked reduction in species richness and the lowest *α*-diversity indices compared to non-infection people with or without *C. difficile* colonization (Wan et al., 2022; Wei et al., 2022; Vázquez-Cuesta et al., 2023). Furthermore, *β*-diversity and co-occurrence network analysis confirmed that the overall microbial community structure of the CDI group was significantly distinct from the other three groups, indicating a distinct microbial architecture associated with infection (Ke et al., 2021). In line with the previous studies (Herrera et al., 2022; Mahnic et al., 2022), the relative abundance of potential probiotics such as *Lactobacillus* and *Bifidobacterium* decreased in CDI, while the relative abundance of *Escherichia*, *Klebsiella* increased.

Previous studies have demonstrated that *C. difficile* colonization can alter the gut microbiota (Couturier et al., 2022; Vázquez-Cuesta et al., 2023), therefore, to identify species which are not merely associated with the presence of *C. difficile*, but specifically linked to the critical transition from colonization to active disease, we subsequently focused our analysis on identifying differentially abundant species between the *C. difficile* asymptomatic carriage (AC) and infection (CDI) groups. Among these specific microbial candidates, *O. splanchnicus* was identified as a key species negatively correlated with disease severity. Notably, a recent study revealed a protective role for *O. splanchnicus* in inflammatory bowel disease (IBD), mediated through its involvement in the conversion of lithocholic acid (LCA), which helps alleviate colitis and maintain intestinal barrier integrity (Xu et al., 2025). Given that CDI is primarily characterized by intestinal infection and barrier disruption, this established mechanism may, at least in part, explain the observed negative correlation between a high abundance of OS and milder CDI symptoms in our cohort. However, it has not been established whether *O. splanchnicus* has a direct inhibitory effect on *C. difficile*.

Several mechanisms of different bacteria for inhibiting *C. difficile* pathogenicity have been described. For instance, Kolling *et al.* demonstrated that *Streptococcus thermophilus* directly suppresses the growth of *C. difficile* as well as the TcdA toxin *in vitro* by secreting lactic acid, which exerts a dose-dependent bactericidal effect (Kolling et al., 2012). Another study utilizing an automated anaerobic *in vitro* fermentor demonstrated that the supernatant of *Clostridium scindens* contains secreted factors which suppress the growth of *C. difficile* and the expression of its TcdA and TcdB toxins (Saenz et al., 2023). In contrast to mechanisms centered on growth inhibition, our experimental results revealed that co-culture with *O. splanchnicus* did not significantly suppress the growth of *C. difficile*, which exhibited robust proliferation between 6 to 18 h. This result was in line with a previous study which employed a culturomics approach to build a strain library for identifying *C. difficile*-inhibiting species (Ghimire et al., 2020), and this research also isolated *O. splanchnicus* and similarly reported no inhibitory effect on *C. difficile* growth, aligning with our observation. Therefore, the combined evidence indicates that the mitigation of *C. difficile* pathogenicity by *O. splanchnicus* is not achieved through restraining the growth of vegetative cells.

We further demonstrated that *O. splanchnicus* suppresses *C. difficile* toxin production especially TcdB toxin in a time-dependent manner, with the effect being most pronounced at the highest initial *O. splanchnicus* ratio (CD: OS = 1:9). The most significant reduction occurred during the stationary phase (48–72 h), which coincides with the peak toxin production in monoculture (Metcalf et al., 2010). Concurrent qPCR analysis revealed that the suppression is initiated at the transcriptional level. A significant downregulation of *tcdB* expression was specifically observed at early and mid time points (12–24 h) in co-cultures with a high initial *O. splanchnicus* ratio. This early transcriptional inhibition provides a direct molecular explanation for the subsequent reduction in toxin protein levels detected later during the stationary phase. However, we found that toxin levels increased in both co-culture groups (CD: OS = 1:1 and 9:1) at 72 h, with the levels showing no statistically significant difference from the *C. difficile* monoculture. And this increase was accompanied by a significant upregulation of *tcdB* gene expression in the CD: OS = 9:1 group. We propose that this late-stage resurgence of virulence is likely due to a decline in the metabolic activity of *O. splanchnicus* as the co-culture enters the late stationary phase. This dynamic aligns with the fundamental ecological principle that a commensal bacterium’s protective function is closely linked to its abundance and activity within its niche (Buffie et al., 2015; Caballero-Flores et al., 2022). For instance, *Clostridium scindens* must reach a sufficient abundance to produce inhibitory secondary bile acids (Studer et al., 2016). Similarly, *O. splanchnicus* likely requires a sustained level of metabolic activity to continuously suppress *C. difficile* toxin production, possibly via nutrient competition.

Concurrently, we observed a notable increase in *C. difficile* sporulation within the co-culture system, particularly evident at the 24-h time point. This phenotypic shift aligns with the well-established regulatory circuitry of *C. difficile*, where toxin production and sporulation are coordinately influenced by environmental cues such as nutrient availability (Majumdar and Govind, 2022; Masset et al., 2024). Co-culture with gut commensals can impose nutrient stress on *C. difficile*, and the consumption of preferred carbon and nitrogen sources by competitors can alter the metabolic environment, thereby accelerating spore formation (Sulaiman et al., 2024; Dong et al., 2025). Therefore, we proposed that *O. splanchnicus* may accelerate the transition of *C. difficile* vegetative cells into dormant spores, which consequently lead to the observed reduction in toxins production. However, both the sporulation-enhancing and toxin-suppressing effects exhibited dynamic changes over time. By the 72-h time point, the difference in spore counts between co-culture groups and the *C. difficile* monoculture was no longer statistically significant. We attribute this convergence, along with the late-stage rebound in toxin production noted earlier, primarily to a decline in the metabolic activity of *O. splanchnicus* as the co-culture enters the late stationary phase. This observation underscores that the anti-virulence effect of OS is not static but depends on its sustained metabolic activity within the ecological niche.

Simultaneously, the potential epidemiological implications of OS-enhanced *C. difficile* sporulation warrant careful consideration. From a transmission perspective, an increase in spore production could theoretically elevate the environmental persistence and spread of *C. difficile* within healthcare settings, as spores are the primary, highly resilient vector for dissemination (Martin et al., 2016; Schnizlein and Young, 2022). However, this potential risk must be evaluated within a more nuanced and holistic framework. Crucially, *O. splanchnicus* concurrently and significantly suppresses toxin production, which directly reduces disease severity and symptomatic diarrhea in the host. Since symptomatic diarrhea is the primary driver of infectious, spore-laden stool output, this reduction could substantially decrease the overall source of environmental contamination (Riggs et al., 2007; Crobach et al., 2018). Furthermore, our simplified *in vitro* model cannot replicate critical *in vivo* factors, such as intestinal peristalsis for physical clearance and a fully functional immune response that profoundly influence the actual shedding, survival, and ultimate transmission potential of spores. Therefore, future studies employing animal infection models and clinical observations are needed to evaluate the actual impact of *O. splanchnicus* supplementation on *C. difficile* colonization, recurrence, and environmental dissemination.

In summary, our studies suggest that *O. splanchnicus* plays a crucial role in mitigating *C. difficile* pathogenesis. We show that higher *O. splanchnicus* levels correlate with milder disease in patients and demonstrate in vitro that it suppresses *C. difficile* toxin production, particularly TcdB, while promoting sporulation at earlier stage. The identification of *O. splanchnicus* provides a novel and promising candidate for next-generation live biotherapeutics. Nevertheless, further studies are needed to define its precise mode of action and effective dose *in vivo* for therapeutic development.

## Data Availability

The datasets presented in this study can be found in online repositories. The names of the repository/repositories and accession number(s) can be found at: https://www.cncb.ac.cn/, PRJCA050088.
