# Supplementary material for: Odoribacter splanchnicus inhibits toxin production in Clostridioides difficile: insights from clinical correlation and in vitro validation
Source: Front Microbiol. 2026 Jan 22;17:1741232. doi: 10.3389/fmicb.2026.1741232 (PMC12872478; doi:10.3389/fmicb.2026.1741232)
Supplement: Supplementary file 1 [file Data_Sheet_1.docx]

Supplementary Material

# Supplementary Figures and Tables

For more information on Supplementary Material and for details on the different file types accepted, please see [here](https://www.frontiersin.org/guidelines/author-guidelines#supplementary-material).

## Supplementary Figures


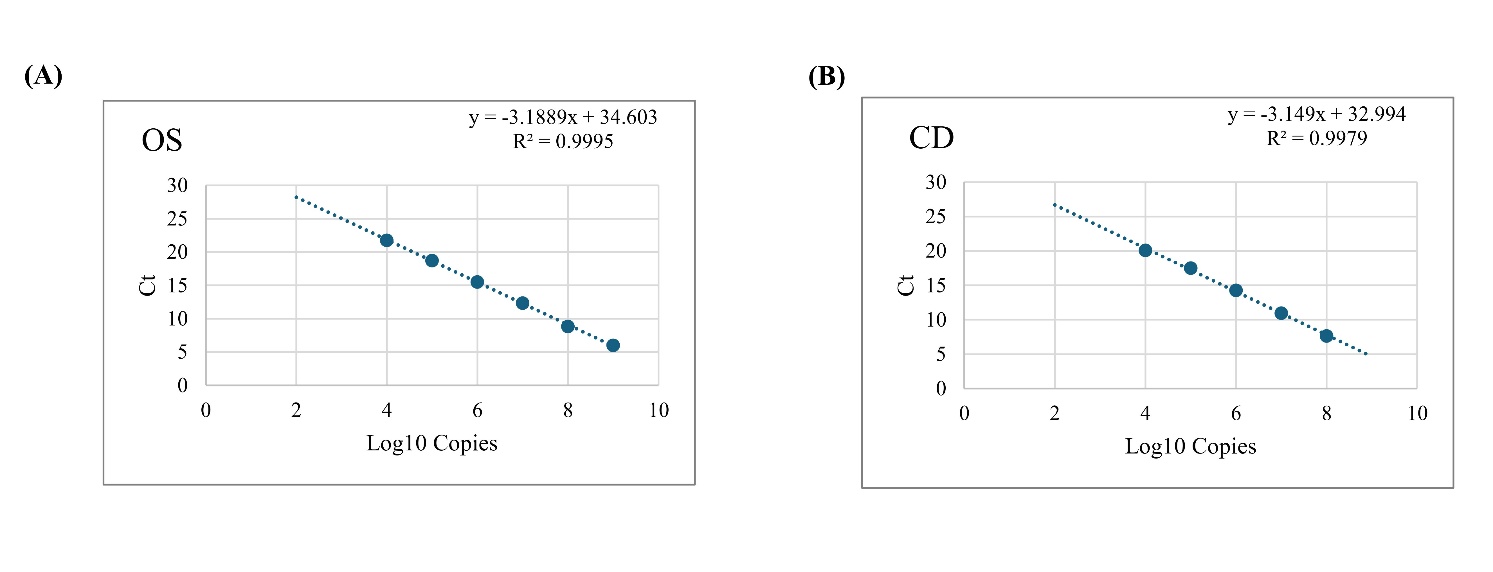
**Supplementary Figure S1.** Standard curve of the real-time quantitative PCR method. (A) Results of the standard positive plasmid of *O. splanchnicus*. (B) Results of the standard positive plasmid of *C. difficile*.


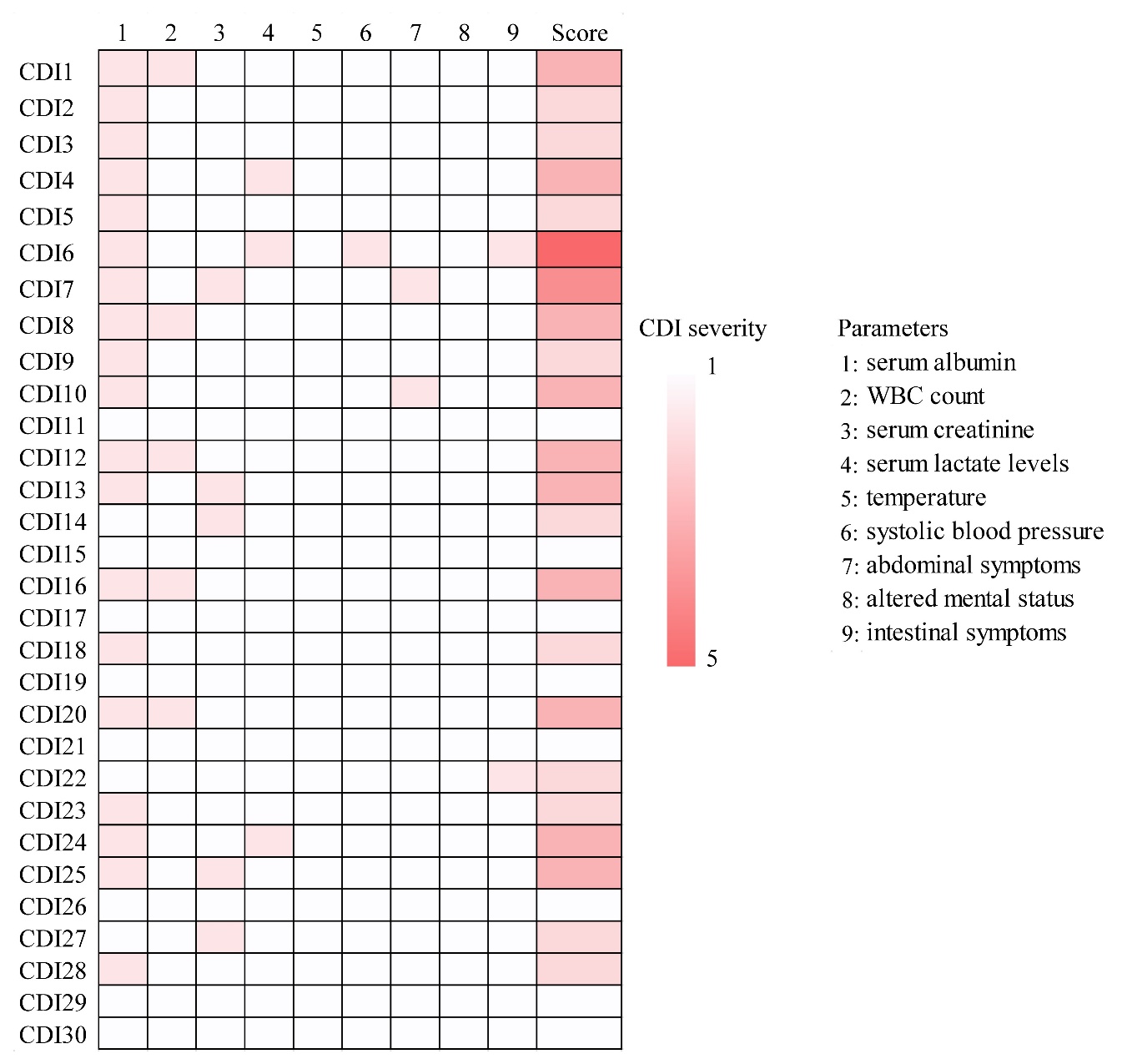


**Supplementary Figure 2.** CDI severity score index of 30 CDI patients.
